# Hybridization-based gold nanoprobe assay for the detection of candidemia in ICU patients - A novel method

**DOI:** 10.6026/973206300222942

**Published:** 2026-05-31

**Authors:** Anitha Subramanian, Anupma Jyoti Kindo, Renuka M.K, Anusha Karunasagar

**Affiliations:** 1 Department of Microbiology, Faculty of Allied Health Sciences, Sri Ramachandra Institute of Higher Education and Research, Porur, Chennai, Tamil Nadu, India; 2 Department of Microbiology, Sri Ramachandra Institute of Higher Education and Research, Porur, Chennai, Tamil Nadu, India; 3 Department of Critical Care Medicine, Sri Ramachandra Institute of Higher Education and Research, Porur, Chennai, Tamil Nadu, India; 4 Department of Microbiology, The Princess Alexandra Hospital NHS Trust, Harlow, United Kingdom

**Keywords:** Candidemia, gold nanoprobe (AuNP), hybridization, illness

## Abstract

Candidemia is a life-threatening infection in ICU patients, with mortality rates of 35-75%, yet current diagnostic methods such as blood culture and conventional Polymerase chain reaction (PCR) lack the sensitivity required for timely detection. Therefore, it is of interest to address this unmet diagnostic need, we developed a hybridization-based gold nanoprobe (AuNP) colorimetric assay for the direct detection of Candida DNA from 3 ml EDTA blood samples. AuNPs were functionalized with Candida-specific thiol-modified oligonucleotide probes using a modified salt-aging process and target DNA hybridization was performed at 56°C followed by visual colour assessment. The assay demonstrated 76% sensitivity, significantly outperforming conventional PCR at 24%, with positive results showing a distinct red-to-purple colour shift and a detection limit of 10 ng/µL. This rapid, low-cost, naked-eye-readable method using UV-Vis spectroscopy is particularly suitable for resource-limited ICU settings, offering a practical tool for earlier candidemia diagnosis and improved clinical outcomes.

## Background:

Candidemia poses a significant mortality risk in ICU patients, with death rates ranging from 35-75% and an incidence of 0.24 to 34.3 per 1,000 ICU hospitalizations [1]. Early start of antifungal treatment is critical, as ICU-acquired candidemia typically affects patients with multiple underlying medical and surgical risk factors, often requiring prolonged medications [2]. Despite advances, blood culture remains the gold standard for detecting invasive candidiasis, but it is time-consuming (up to 48 hours) and may miss early or low-level infections [3]. PCR inhibitors like hemin and EDTA can lead to false negatives. Due to low Candida DNA levels, more sensitive detection methods are needed [4]. To address these challenges, nanomaterials are used as signalling probes for DNA detection. Commonly used nanomaterials in biosensing include quantum dots, carbon nanotubes, graphene, polymeric nanomaterials and metal and metal oxide nanoparticles. Among these, gold nanoparticles are ideal for colorimetric sensing due to their optical properties, adjustable size and surface plasmon resonance effects [5]. In the traditional salt-aging method by Mirkin *et al.*(1996), NaCl was added gradually to stabilize probe attachment for 2 days [6]. The functionalization process involves thiolated single-stranded oligonucleotides, which require reduction with dithiothreitol (DTT), tris (2-carboxyethyl) phosphine (TCEP) or mercaptoethanol, followed by purification [7]. However, our approach introduces a novel modification to the salt-aging process, significantly reducing the time required to break the probe's disulfide bonds and simplifying the functionalization process the shorter process and improved efficiency support rapid assay development. Colloidal gold nanoparticles (AuNPs) are widely used with alkyl-thiol-modified DNA for stable conjugation [8]. Therefore, it is of interest to develop a hybridization technique for detecting Candida in blood using gold nanoparticles functionalized with Candida-specific oligonucleotide probes.

## Methodology:

## Study design and setting:

It is a retrospective observational diagnostic study. A fixed volume of 3 mL of EDTA blood was collected and stored at 4°C. EDTA blood samples were obtained within 12 hours of the time the patient's actual positive blood was drawn. Candida albicans ATCC 90028 was used as a control strain.The study was done after obtaining Institutional Ethics Committee approval.

## Participants:

A total of 80 EDTA blood samples from ICU patients were analysed. Sample size estimation indicated that 50 diseased subjects were required to estimate sensitivity (87.5%, precision ± 8 %) and 30 subjects for specificity (99%, Precision ± 3%) at a 95% confidence level. Patients whose blood cultures were positive for Candida and who had ICU admissions lasting more than 5 days were included. Patients with known Candida culture positivity before ICU admission or with an ICU stay of less than 5 days were excluded.

## Standardization of DNA isolation from spiked blood and patient samples:

## Spiked blood samples:

Candida albicans ATCC 90028 cells were serially diluted (100 to 106 CFU/ml) and 200 µL of each dilution was mixed with 3 ml of EDTA blood collected from healthy patients [9]. DNA was extracted from these spiked blood samples and tested using the same AuNP oligo probe assay to assess its sensitivity in the presence of inhibitors. These two approaches allowed assessment of both the analytical limit of detection and the clinical detection potential of the hybridization assay. DNA extraction was performed using the QIAamp® DNA Blood Mini kit(Qiagen).

## Patients' EDTA blood sample:

The same protocol was followed for processing the patients' EDTA blood samples. Briefly, RBC lysis buffer (ammonium chloride (NH4Cl) and sodium bicarbonate (NaHCO3)) was added to 3 mL of EDTA blood, followed by incubation at room temperature for 10 minutes. Centrifugation at 1800 rpm for 10 minutes separated the supernatant and the process was repeated until a visible white precipitate formed. PBS (200 µL) was added. DNA extraction was performed according to the kit's instructions. DNA concentration was measured at 260 nm using a nanophotometer and stored at -20°C.

## Polymerase chain reaction (PCR):

The amplification of ITS1 (5'TCC GTA GGT GAA CCT GCG G-3') and ITS4 (5'TCC TCC GCT TAT TGA TAT GC-3') [10]. The target was amplified using a master mix containing 12.5 µL of PCR mix (GeNei, Bangalore), 0.5 µL each of ITS 1 and ITS4 primers (Sigma), 2µLof template DNA and the total volume was made up to 25 µL with nuclease-free water. The thermocycler (Thermo Fisher, Veriti) was programmed as follows: initial denaturation at 950 °C for 7min, denaturation at 950°C for 30 sec, annealing at 560 °C for 30sec, extension at 720°C for 30 sec and final extension at 720 °C for 5 min. The PCR product was electrophoresed in a 1.5% agarose gel and visualized under a UV transilluminator.

## Gold nanoparticles (AuNP):

The AuNP was obtained from Sigma as a stable suspension in a citrate buffer at 20nm (Sigma). The polydispersity index (PDI) was <0.2 and the maximum absorbance ranged from 518 to 522nm, consistent with surface plasmon resonance typical of well-dispersed AuNPs. The particle concentration was confirmed to be between 5.89E+11 and 7.19E+11 particles/ml and the optical density (OD) was 1. The colloidal gold nanoparticle was stored at 4°C until use.

## Probe design:

The Candida-specific sequence targeting the ITS (Internal Transcribed Spacer) gene region, a repeat unit of rRNA, was obtained from the NCBI database and the probe was designed using Primer3 (https://primer3.ut.ee). The final 20-base probe sequence, 5'-CAGCGGTTTGAGGGAGAAAC-3', was purchased from Sigma. The thiol modifier was attached at the 3'end of the oligonucleotide probe to enable conjugation with colloidal gold nanoparticles.

## AuNP-oligoprobe preparation:

**Figure 1:**
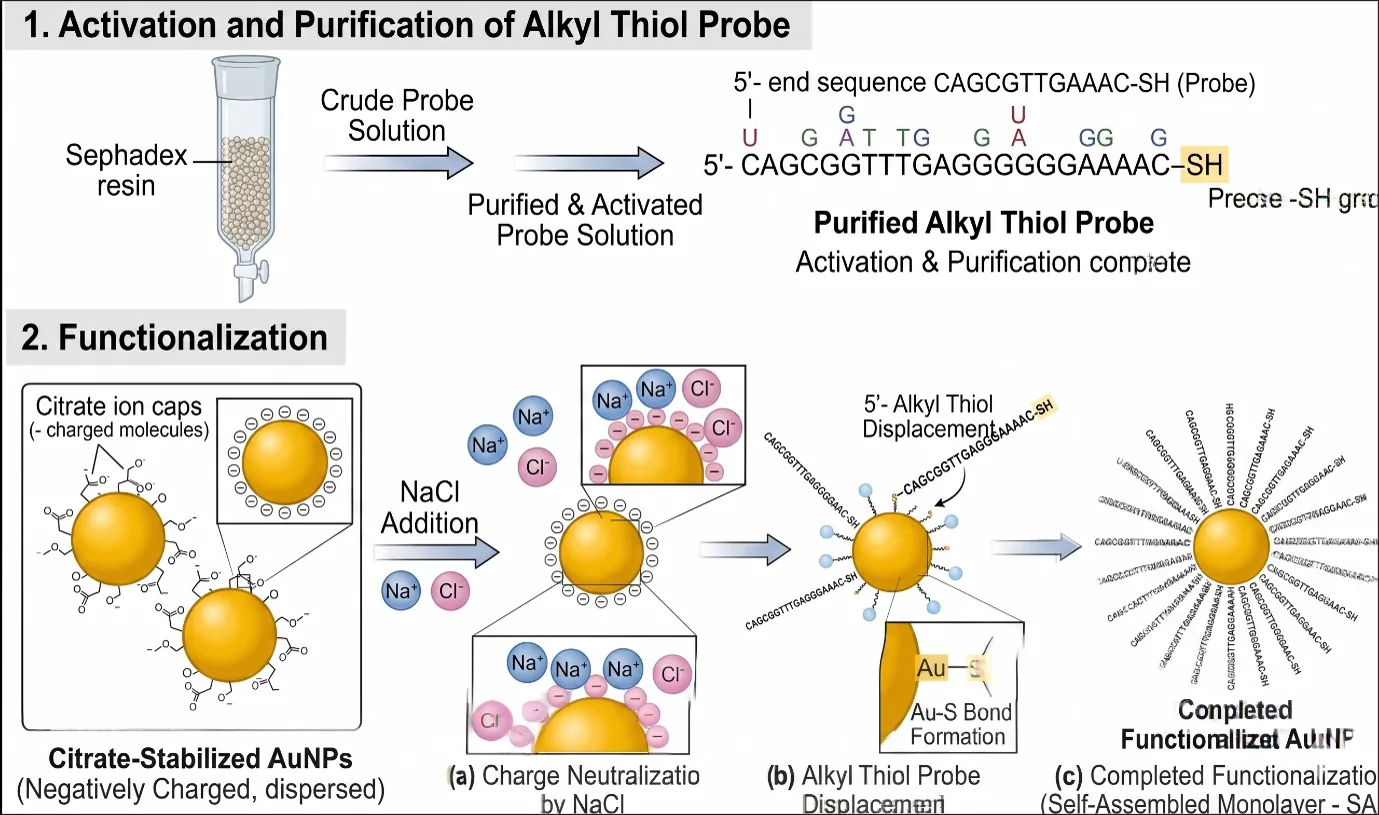
Functionalization of the probe steps

The AuNP-oligo probe was prepared using the salt-aging method by Mirkin *et al.*with modifications [6]. A 0.5 µL aliquot of the probe (100 mM) was incubated for 1 hour at room temperature (RT) with 0.5 µL 2-mercaptoethanol in 100 µL of the phosphate buffer (10 mM, pH 8). Following incubation, it was filtered through a Sephadex G-25 column for 3 min (Figure 1). A nanophotometer (Implen, Germany) was used to measure the eluate to determine the probe's concentration. The probe concentration was adjusted to 2 ng/µL.

## Standardization of the functionalization of the probe:

200µL of AuNP was incubated with 1µL of the processed thiol-modified oligonucleotide probe (1:200 ratios) as per Wu *et al.*[11]. 50mM, 100mM and 200mM salt solutions were prepared in phosphate buffer (NaCl in 10 mM PBS, pH 8). Every hour, NaCl was progressively added, starting from the lowest to the highest concentration and the samples were centrifuged for 10 minutes at 8000Xg. After discarding the supernatant, the phosphate buffer was added. The completely functionalized AuNP-oligo probes maintain the same colour as the unmodified AuNPs (red) without any visible aggregates. It was then stored in the dark at room temperature until use. The reactions during NaCl addition are shown in (Figure 1).

## Characterization of functionalized AuNP:

Numerous techniques are available to analyze nanoparticle properties. Scanning and Transmission electron microscopy (SEM/TEM), atomic force microscopy (AFM) and X-ray diffraction (XRD) require the sample to be in a dry, powdered form, limiting their applicability. Phase analysis light scattering (PALS) is a solution-based technique used to measure zeta potential, providing insights into the surface charge and colloidal stability of nanoparticles in suspension [11, 12].

## AuNP-oligo probe-Hybridization assay:

The temperature needed for the hybridization protocol was standardized with Candida albicans ATCC 90028. Positive control: Candida DNA; negative control: bacterial DNA; PBS was used as the blank control. To determine the minimum amount of Candida DNA detectable by the assay under ideal, inhibitor-free conditions, known concentrations of purified Candida albicans ATCC 90028 DNA (106 ng/µL to 0 ng/µL) were prepared in 10 mM PBS buffer. Each dilution was tested in the AuNP-oligo probe assay, and the colour was noted to establish the assay's baseline sensitivity.

## Statistical analysis:

A 2x2 contingency table analysis was used to compare the performance of the hybridization assay and PCR against blood culture results [13].

## Results:

**Figure 2:**
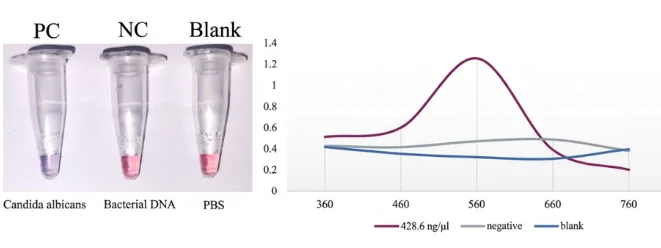
Optimization of the AuNP-oligo probe hybridization assay

**Figure 3:**
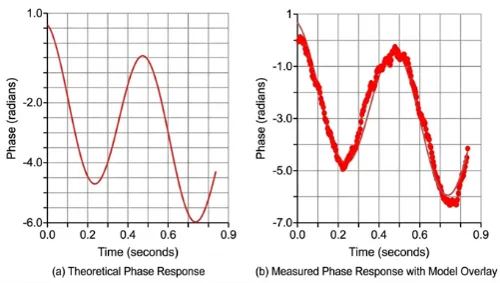
Phase plots obtained using PALS

**Figure 4:**
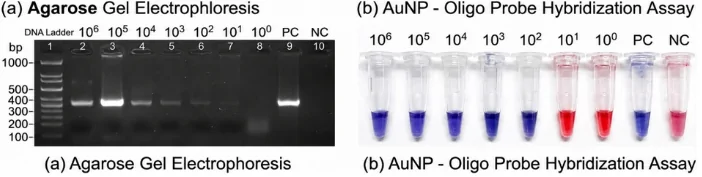
Results of Agarose gel electrophoresis and AuNP-oligo hybridization assay

**Figure 5:**
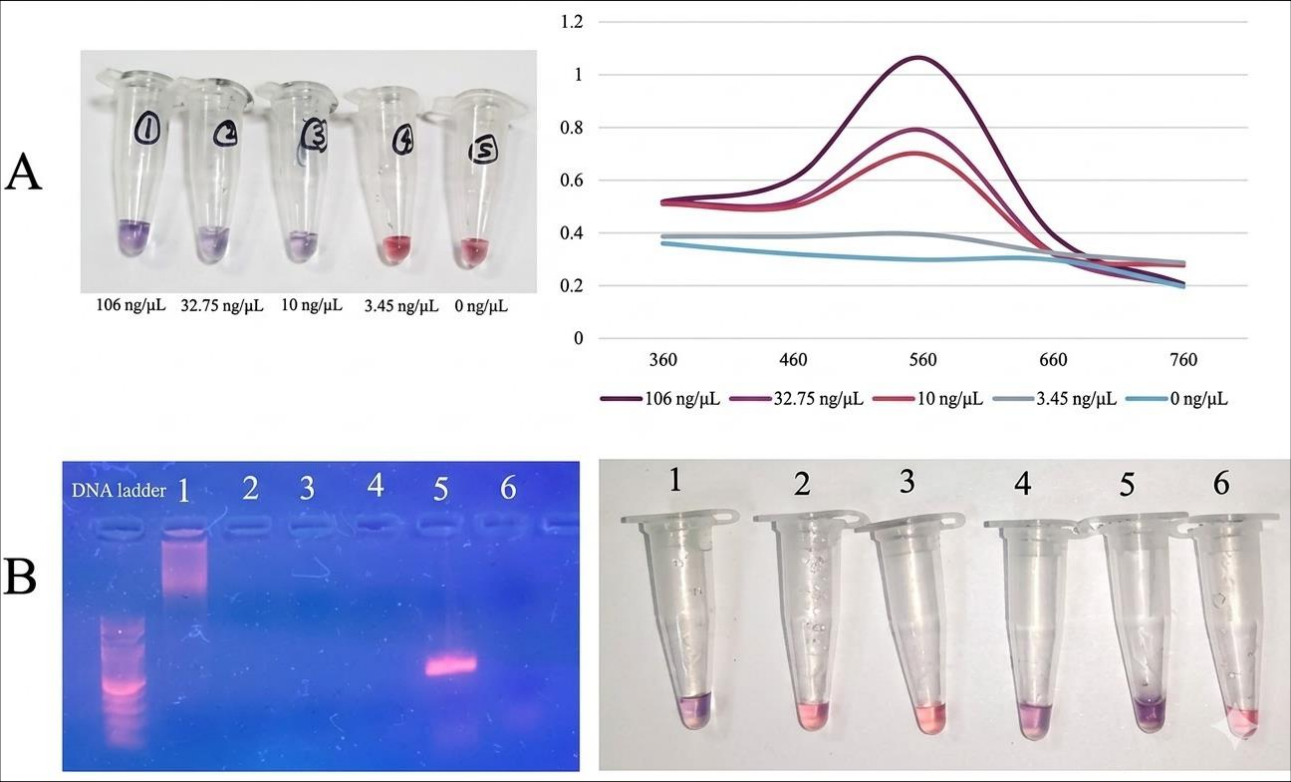
Sensitivity of AuNP - oligo probe hybridization assay and AuNP-oligo probe hybridization assay's specificity on Patients' blood samples

It was observed that annealing at 56°C was the optimum temperature for hybridization of the AuNP-oligo probe with the target DNA. Positive control (PC), Candida DNA shows a purple colour, while the negative control (NC) (Bacterial DNA) and blank (PBS buffer) remain red (Figure 2). UV-vis spectroscopy confirms target DNA detection in the positive control, with maximum absorbance. The sample cell was rinsed with 1 mM potassium nitrate (KNO3) before measurement. A low polydispersity index (PDI) of 0.320 indicates a narrow size distribution and high nanoparticle uniformity. Zeta potential was -16.89 mV using Smoluchowski analysis type (Centre for Nanobiotechnology, Vellore Institute of Technology) with electrophoretic mobility of -1.32 (µ/s)/(V/cm). Sinusoidal phase plots confirmed measurement stability (Figure 3). PCR of Candida-spiked blood (106-103 CFU/ml; lanes 2-5) yielded bands, but lower loads (<102 CFU/ml; lanes 6-10) were undetected. In contrast, AuNP-oligo hybridization assay produced a purple color and corresponding absorbance peaks even in PCR-negative samples (102), indicating higher sensitivity of the nanoprobe-based detection method (Figure 4). The assay demonstrated sensitivity across DNA dilutions, with visible colour changes, detecting Candida DNA even at low concentrations (10 ng/µL) (Figure 5). Absorbance peaks correlated with DNA levels. Purple colour in tubes 1, 4 and 5 confirmed nanoprobe aggregation, indicating Candida presence, while red showed its absence (Figure 5). In a representative culture, positive patient samples, PCR showed no bands, whereas hybridization yielded positive results, confirming the superior sensitivity of the AuNP-oligo assay over conventional PCR. A total of 80 EDTA blood samples were analyzed. The statistical analysis compared hybridization and PCR gel electrophoresis with blood culture results for the detection of Candida in EDTA blood samples. Hybridization exhibited higher sensitivity (76%) compared to PCR (24%), while PCR showed better specificity (76.7%) than hybridization (53.3%). Among 50 blood culture-positive cases, hybridization detected 38 positives, of which PCR confirmed 12. Of 30 culture-negative cases, hybridization identified 14 positives, of which 7 were PCR-positive. To ensure consistency and reproducibility of the results, the method was repeated with multiple replicates.

## Discussion:

The proposed method offers a significant reduction in assay time, with 3 hours for probe functionalization and 17 minutes for hybridization. Careful standardization of the incubation period and the concentrations of salt and 2-mercaptoethanolwere optimized. In the functionalization process, mercaptoethanol was found to be suitable for reducing the disulfide bonds in the oligonucleotide used in this study. This study aims to amplify Candida DNA from blood samples using conventional PCR to generate target DNA for hybridization with gold nanoprobe-based oligonucleotides. Colloidal AuNPs are used in various bio detection schemes. Padmavathy *et al.*(2012) developed a gold nanoprobe tool for detecting Escherichia coli DNA in urine samples [12]. Recently, Clack *et al.*(2024) developed a colorimetric method for detecting Candida albicans that can be observed with the naked eye [14]. A desalting step is an important challenge in removing impurities that can lead to aggregation during the functionalization of AuNPs [6]. To overcome this, a Sephadex column was used to purify the alkyl thiol probe, removing mercaptoethanol, which is responsible for the aggregation of AuNPs upon mixing with the activated alkyl thiol probe. Various methods, such as salt aging, vacuum centrifugation, BSPP (bis (p-sulfonatophenyl) phenylphosphine dehydrate dipotassium salt) coating, Tween 80 and functionalization of AuNPs with thiol-DNA, differ in components and time [15]. This study presents a significant innovation by modifying the traditional salt-aging process to dramatically reduce the functionalization time of the AuNP-probe conjugate. To produce effective Au nanoprobes for diagnostic assays, it is crucial to balance the oligonucleotide ratios. These ratios must be high enough to ensure the colloidal stability of the Au nanoprobe while remaining low enough to facilitate efficient hybridization with the DNA target. The AuNP (OD1): oligonucleotide ratio used was 200 µL: 2 ng/µL; the solution remained stable upon NaCl addition. A ratio exceeding 2 ng/µL led to aggregation, especially when salt concentrations were above 200 mM. During standardization, thorough optimization was conducted to determine the optimal probe and NaCl concentrations for a stable Au nanoprobe. The stability of the Au nanoprobe depends on the optimal concentrations of the probe, AuNP and NaCl and the concept of attaching the probe in less time was accordingly adapted [16]. The Na+ ions enable the alkyl thiol probes to approach and attach to the gold nanoparticle surface more easily.The culture-positive blood sample showed a PCR-negative but hybridization- positive result, highlighting the higher sensitivity of the AuNP assay. At the same time, traditional agarose gel electrophoresis failed to detect Candida DNA, possibly due to low DNA concentrations or PCR inhibitors. The colorimetric change observed in the AuNP-oligo assay indicates that the hybridization method can detect even minimal amounts of target DNA that might be overlooked by conventional PCR [17]. This result demonstrates the potential of the hybridization technique as a reliable alternative to PCR. However, the reported ranges for specificity and sensitivity across various studies are 54% to 100% and 56.2% to 100%, respectively, using a range of target primers and PCR techniques [1]. To the best of our knowledge, this method is being used for the first time to detect Candida in EDTA blood using the hybridization technique. Detecting DNA concentrations below 20ng in agarose gel electrophoresis is challenging [18], while the hybridization technique detects around 10ng of DNA. The blood volume available for PCR was limited to 3ml, which is lower than the 5-10ml used for blood cultures, reducing PCR yield. PCR exhibited inconsistencies with some initially positive samples turning negative upon retesting [19]. The sample size was relatively small and from a single centre, which may affect the broader applicability of the finding. Blood culture, used as the reference standard, has limited sensitivity (∼50-70%) and may underestimate the molecular method's performance.

## Conclusion:

Timely diagnosis of invasive candidiasis, potentially reducing mortality and optimizing antifungal therapy is relevant. The simplicity and rapid turnaround of this method make it suitable for point-of-care testing in intensive care units.

## Advancement to knowledge:

This study introduces the first reported application of a hybridization-based gold nanoprobe (AuNP) assay for the direct detection of Candida DNA from EDTA blood in ICU patients with candidemia. A key innovation is the modified salt-aging functionalization protocol that reduces probe preparation time from 2 days to 3 hours, without compromising probe stability or assay performance. The colorimetric assay demonstrated superior sensitivity (76%) compared to conventional PCR (24%), detecting Candida DNA at concentrations as low as 10 ng/µL-including in PCR-negative culture-positive samples. This simple, low-cost, rapid and naked-eye-readable method offers a practical molecular diagnostic alternative for resource-limited ICU settings, with the potential to improve early detection and antifungal treatment initiation in critically ill patients.

## Financial support and sponsorship:

Nil

## Ethics approval: 

The study was conducted after obtaining Institutional Ethics Committee approval, with reference number IEC/20/FEB/157/28.
